# Prise en charge des patients diabétiques au cours du jeûne du Ramadan: application des recommandations internationales en pratique clinique

**DOI:** 10.11604/pamj.2020.36.316.7201

**Published:** 2020-08-21

**Authors:** Saloua Elamari, Siham Elaziz, Asmaa Chadli, Ahmed Farouqi

**Affiliations:** 1 Service d’Endocrinologie du CHU Ibn Rochd, Casablanca, Maroc

**Keywords:** Jeûne, diabète type 2, Ramadan, équilibre métabolique, recommandations internationales, Fasting, type 2 diabetes, Ramadan, international recommendations, metabolic balance

## Abstract

Le jeûne n’est pas sans risques chez les patients diabétiques. L’objectif de notre étude est d’évaluer l’impact du jeûne chez une cohorte de patients diabétiques ayant bénéficié d’une préparation au jeûne avec ajustement des traitements selon les recommandations de l’association américaine du diabète (ADA) en 2010. Il s’agit d’une étude prospective de cohorte menée en 2014, incluant des patients diabétiques désirants jeûneer au cours du mois de Ramadan consentant pour participer à l’étude. Nous avons exclu les patients considérés comme à très haut risque selon la classification de l’ADA. Les patients ont bénéficié d’une consultation avant le mois du Ramadan (J0) comprenant éducation et ajustement du traitement, suivi d’une consultation au cours du Ramadan (J7) puis à J30 et à J60. Notre étude a inclus 34 patients, dont 60% sont des femmes, d’âge moyen de 50,4 ans avec une ancienneté moyenne du diabète de 6,2 ans et un index de masse corporelle (IMC) moyen de 27,83 kg/m^2^. Les ajustements thérapeutiques réalisés (J0) étaient une diminution de la dose de sulfamides hypoglycémiants (SH) et la mise sous gliptine chez les patients à risque hypoglycémique, ainsi qu’une répartition de la dose de metformine. Au cours de la première semaine (J7), deux patients ont présenté une glycémie inférieure à 0,7g/l avant la rupture du jeûne, 38% de l’échantillon ont présenté une glycémie >2g/l après la rupture du jeûne. Nous avons noté des erreurs diététiques chez 15% des patients. L’ajustement à (J7) a consisté à adapter la dose du SH ou à ajouter une gliptine. Aucun patient n’a présenté d’hyperglycémie majeure, de cétose ou d’hypoglycémie grave. Une patiente a présenté une arythmie complète par fibrillation atriale (ACFA) sur cardiopathie ischémique méconnu avec arrêt du jeûne. Trois patients ont arrêté le jeûne entre J15 et J20 devant une asthénie intense sans déséquilibre glycémique. La moyenne d’HBA1C chez les patients après le jeûne était de 7,10 versus 6,8% avant le jeûne (p=0.42). Les facteurs significativement associés au déséquilibre glycémique après le jeûne avec des HBA1C > à 8% étaient principalement les taux d’HBA1C de départ (p=0.002), l’absence d’autosurveillance (p=0.01), et l’ancienneté du diabète (p=0.06). Une bonne évaluation du niveau de risque du patient, une éducation thérapeutique, une autosurveillance glycémique et un ajustement du traitement selon les recommandations internationales, permettent aux patients diabétiques musulmans de diminuer le risque lié au jeûne. Néanmoins une consultation au cours du Ramadan (J7) permet une correction des erreurs diététiques et un ajustement thérapeutique si cela est nécessaire.

## Abstract (en)

Fasting is not without risk in diabetic patients. The aim of our study was to assess the impact of fasting on glycémic control in diabetic patients prepared for fasting as recommended by the American Diabetes Association (ADA) in 2010. We conducted a prospective cohort study in 2014, including diabetic patients wishing to fast during Ramadan, consenting to take part in this study. Were excluded patients considered at very high risk according to ADA classification. Patients included had a consultation before the month of Ramadan (D0) consisting of education and treatment adjustment. This was followed by a medical visit during Ramadan (J7), then at J30 and J60. Results analysis was done using SPSS Smartviewer15.0 Our study included 34 patients, of whom 60% were women, with a mean age of 50.4 years, an average 6.2-year diabetes history, and a mean body mass index of 27.83kg/m^2^. Therapeutic adjustments (D0) were: decrease dose of sulphonylurea (SH), gliptine add in patients at risk of hypoglycaemia, and a metformin dose distribution. During the first week (D7) two patients had a lower hypoglycemia 0.7g/l before breaking the fast and 38% of the sample showed hyperglycemia great than; 2g/l after breaking the fast. We noted dietary errors in 15% of patients. Adjusting at J7 was to modify the dose of SH or to add a gliptine. No patient showed major hyperglycemia, ketosis or severe hypoglycemia. One patient presented an atrial fibrillation on unknown ischemic cardiopathy with acease of fasting. Three patients gave up fasting between J15 and J20 due to an intense asthenia without hyperglycemia. Mean HbA1c in patients after the fasting was 7.10 versus 6.8% before fasting (p = 0.42). Factors significantly associated to poor glycemic control were mainly HbA1C rate at admission (p = 0.002), absence of self-monitoring (p = 0.01) and diabetes duration (p = 0.06). This study allowed us to show that a good evaluation of diabetic risk level, an education, a glycemia monitoring and an adjustment of the treatment by implementing international recommendations to allow fasting for Muslim diabetic patients with low risk of acute complications and metabolic disorders. A medical visit during the month of Ramadan (D7) would allow for correction of the dietetic mistakes and adjustment of oral agents if necessary.

## Introduction

Le jeûne du mois de Ramadan est un rite religieux représentant l’un des cinq piliers de l’Islam. C’est une obligatoire pour tous les musulmans adultes et en bonne santé. Il a lieu pendant le neuvième mois du calendrier hégirien qui est un calendrier lunaire. Sa durée est donc de 29 ou 30 jours et la durée quotidienne du jeûne peut varier entre 12 et 20 heures en fonction de la saison et du lieu géographique [1]. Il est permis au cours du jeûne de manger et de boire entre le coucher du soleil et l’aube. C’est un jeûne intermittent dans une ambiance festive qui ne s’accompagne pas nécessairement d’une restriction calorique [2]. Cette situation expose les patients diabétiques à une détérioration du contrôle métabolique et à des complications aigues: l’hypoglycémie, l’hyperglycémie, l’acidocétose diabétique ainsi qu’à l’augmentation du risque thromboembolique. Selon l’étude EPIDIAR seulement 62% des patients DT2 et 68% des patients DT1 reçoivent un avis spécialisé et un ajustement thérapeutique durant le mois de Ramadan [3]. A partir de ces constatations plusieurs propositions de prise en charge du diabétique pendant le Ramadan ont été publiées, nous citons les recommandations américaines (American Diabetes Association, ADA) publiées en 2010 [4] et asiatiques (South Asian Consensus Guideline) [5], ainsi que les propositions thérapeutiques d’Arabie Saoudite [6]. L’objectif de notre étude est d’évaluer l’impact du jeûne sur des paramètres anthropométriques notamment le poids, sur la tension artérielle (TA) systolique et diastolique et sur l’équilibre glycémique (HBA1C) chez des patients préparés au jeûne selon les recommandations internationales par un ajustement thérapeutique et une séance d’éducation sur la diététique et la conduite du jeûnee.

## Méthodes

Il s’agit d’une étude prospective incluant tout patient diabétique suivi au service d’endocrinologie désirant jeûneer le mois de Ramadan 2014 consentant pour participer à l’étude. Nous avons exclu les diabétiques à très haut risque soit les diabétiques type 1 ou type 2 sous insuline, les diabétiques récemment découvert (<3 mois) ou ayant un épisode récent (<3 mois) d’hypoglycémie sévère, d’acidocétose ou de coma hyperosmolaire, les patients insuffisamment contrôlés jugé sur des taux HbA1c supérieur à 9% ou des hypoglycémies fréquentes ou non ressenties ainsi que les patients ayant un diabète instable (épisodes d’hypo- ou hyperglycémie au moins 3 fois par semaine), les sujets âgés vu le risque de réhydratation, la démence ou les troubles de la vigilance. Les patients ayant une hypertension artérielle sévère non contrôlée (TA > 170mmhg et/ou 100mmhg) ou une maladie rénale chronique (clairance de la créatinine < 60 ml/min) ainsi que la présence d’un tableau clinique d’insuffisance hépatocellulaire, d’un angor instable ou d’une insuffisance cardiaque.

**Déroulement de l’étude:** les patients ont consulté en pré Ramadan (J0) avec recueil des caractéristiques démographiques et cliniques: âge, sexe, poids, index de masse corporelle (IMC) et tension artérielle moyennant un tensiomètre à mercure manuel. Ils ont bénéficié d’une éducation diététique en préconisant un apport normo glucidique, normo protidique et hypo lipidique en en 2-3 repas après la rupture du jeûne gras et sucrés au Ftour et en privilégiant les repas riches en sucres complexes au Shour. Un apport hydrique à 1,5 l/nuit au minimum a été conservé. L’activité physique était à éviter avant le Ftour et à privilégier 2 heures après. Les prières de Tarawih sont considérées dans le programme d’exercice physique quotidien. L’intérêt de l’autosurveillance glycémique a été expliqué au patient avec rupture du jeûne si GC < 0,7g/l ou GC > 3g/l [4]. Les traitements ont été ajustés en fonction de l’HBA1C et du risque hypoglycémique du patient selon les recommandations internationales. Les patients sont revus en consultation au cours de la deuxième semaine du Ramadan (J7) afin d’évaluer le déroulement du jeûne (recueil alimentaire, activité physique, carnet d’auto surveillance) avec un éventuel ajustement thérapeutique. Les patients sont revus à la fin du Ramadan (J30) avec prise de leur poids et mesure de leur tension artérielle. L’HBA1C a été faite à 2 mois (J60). Les données ont été recueillies et l’analyse statistique s’est faite via le logiciel SPSS Smartviewer 15.0. Les variables quantitatives sont exprimées en moyenne ± écart-type et les variables qualitatives en pourcentages. Une valeur p < 0,05 est considérée significative.

## Résultats

**Les caractéristiques des patients:** notre étude a inclus 34 patients dont 60% de sexe féminin ayant un âge moyen de 50,4 ± 9,11 ans, et une ancienneté moyenne du diabète de 6,2 ± 4,2 ans. Leur poids moyen est de 78 ± 12,5 kg et leur indice de masse corporel moyen est de 27,8 ± 3,02 kg/m^2^. Vingt pourcent des patients étaient hypertendus équilibrés sous traitement médical, la tension artérielle systolique moyenne était de 137,9 ± 12,43 mmhg et diastolique moyenne de 86,7 ± 7,22 mmhg. Tous nos patients avaient déjà jeûné: quatre ont des antécédents d’hypoglycémies objectivées et huit des antécédents d’hyperglycémies au cours du mois de Ramadan (Tableau 1). Sur le plan thérapeutique 43% étaient sous monothérapie, 50% sous bithérapie et 7% sous trithérapie. Vingt deux patients soit 73% étaient sous sulfamides hypoglycémiants dont 55% sous gliclazide et 45% sous glimépiride à des doses différentes. Nous avons réduit la dose du sulfamide chez 5 patients et nous avons remplacé les sulfamides par une gliptine chez 2 patients. Nous avons fractionné la dose de metformine chez 25 patients soit 2/3 au ftour,1/3 au Shour. L’HBA1C moyenne est de 6,8% ± 0,92. Trente-six pourcent des patients avaient une HBA1C >7%. Le Tableau 1 résume les caractéristiques des patients à l’inclusion dans l’étude.

**Tableau 1:** caractéristiques des patients à l’inclusion

	Moyenne ± écart type ou n (%)
**Caractéristiques des patients (N=34)**	
Age (ans)	50,40 ± 9,11
Sexe feminin (n)	18,00 (58)
Ancienneté du diabète (ans)	6,20 ± 4,20
Indice de masse corporel (kg/m^2^)	27,83 ± 3,02
Hypertension artérielle (n)	7,00 (20)
Hémoglobine glyquée (%)	6,80 ± 0,92
**Traitement**	
Monothérapie	14,00 (44)
Bithérapie	15,00 (50)
Trithérapie	2,00 (6)
Metformine	25,00 (80)
Glimépiride	10,00 (32)
Gliclazide	12,00 (38)
Gliptine	2,00 (6)
**Ajustement du traitement**	
Réduction de la dose de sulfamides	5,00 (14)
Remplacement des sulfamides par une gliptine	2,00 (5)
Fractionnement de la dose de Metformine	25,00 (73)

### Déroulement du jeûne dans notre cohorte

**Observance du jeûne:** dans notre cohorte 30 patients ont observé le jeûne soit 89%. Quatre ont arrêté: une patiente devant une arythmie complète par fibrillation atriale (ACFA) sur cardiopathie ischémique et trois devant une asthénie avec incapacité à continuer le jeûne entre J15 et J20 du Ramadan.

**Complications aigues au cours du jeûne:** deux patients ont présenté une hypoglycémie mineur à 0,7g/l avant lftour. Nous avons diminué la dose de glimipéride chez un patient et changer le glimépiride par du gliclazide chez un patient. Sept de nos patients ne se sont pas auto surveillés par mesure de la glycémie capillaire au cours du jeûne. Aucun patient n’a présenté d’hyperglycémie majeure, de cétose ou d’hypoglycémie grave.

**Hyperglycémie objectivée à l’autosurveillance:** au cours de la première semaine du jeûne 12 patients soit 38% de l’échantillon ont présenté une hyperglycémie > 2g/l après la rupture du jeûne. Des écarts diététiques avec abus de sucres lents et de gras ont été notés chez 15% des patients. Nous avons augmenté la dose de sulfamides chez 6 patients et ajouter une gliptine chez 2 patients, une correction des écarts diététiques a suffi pour corriger l’hyperglycémie chez 4 patients.

**Paramètres cliniques et glycémiques après le mois du jeûne:** après le mois du jeûne le poids moyen des patients étaient de 78,13kg ± 12,2 versus 78kg ± 12,5 (p=0,96). La tension artérielle systolique moyenne était de 136,16 ± 12,55 mmhg (p=0,58). La tension artérielle diastolique moyenne était de 92,36 ± 11,27 mmhg (p=0,02). La moyenne d’HBA1C chez les patients après le jeûne était de 7,10 ± 0,88% versus 6,8 ± 0,9% (p=0,42) (Tableau 2). Nous avons analysé les caractéristiques des patients ayant déséquilibrés leur diabète en post Ramadan (HBA1C >8%). Les facteurs significativement associés au déséquilibre glycémique étaient principalement les taux d’HBA1C élevé à l’inclusion (p=0,002), l’absence d’autosurveillance (p=0,01), et l’ancienneté du diabète (p=0,05) (Tableau 3).

**Tableau 2:** x00E9;volution des paramètres cliniques et glycémiques après le mois du jeûne

Paramètres	Avant Ramadan, Moyenne ± écart type	Après Ramadan Moyenne ± écart type	P
Poids (kg)	78,00 ± 12,50	78,10 ± 12,27	0,96
Tension artérielle systolique (mmhg)	137,90 ± 12,43	136,60 ± 12,55	0,58
Tension artérielle diastolique (mmhg)	86,66 ± 7,22	92,33 ± 11,27	**<0,05**
Hémoglobine glycosylée (%)	6,80 ± 0,92	7,10 ± 0,88	0,42

**Tableau 3:** les facteurs associés au déséquilibre glycémique en post Ramadan

	HBA1C < 8% (n=21), Moyenne ou n	HBA1C ≥ 8% (n=9), Moyenne ou n	P
Age (ans)	51,15	52,85	0,51
Ancienneté du diabète	5,68	7,86	**0,06**
Sexe feminin	14,00	6,00	0,90
Indice de masse corporel (kg/m^2^)	27,50	27,37	0,87
Hémoglobine glyquée à l'inclusion (%)	6,52	7,86	**<0,05**
Absence d'autosurveillance glycémique (n)	4,00	3,00	**<0,05**
Variation du poids au cours du jeûne	-0,05	+ 0,57	0,42

## Discussion

Les patients diabétiques désirent fortement jeûner le mois de Ramadan malgré la tolérance de la religion et la dispense accordée par le Coran à toute personne malade ou susceptible d’être fragilisée par le jeûne [7], d’où l’intérêt du suivi et de l’accompagnement des patients.

**Complications métaboliques aigues:** nous n’avons pas noté d’hypoglycémie sévère de cétose ou d’acido-cétose ou de coma hyperosmolaire. Nos résultats rejoignent ceux d’autres études, portant sur des patients DT2, dans lesquelles le jeûne du mois de Ramadan ne s’est pas accompagné d’une majoration du risque de survenue de complications métaboliques aigues surtout en présence d’une préparation au jeûne [8-15]. Les hypoglycémies graves sont rapportées chez les patients DT2 sous insuline et sous sulfamides hypoglycémiants surtout dans des études observationnelles [16,17]. Le risque hypoglycémique est d’autant plus important chez les patients avec neuropathie végétative et anomalies de la contre régulation [18,19].

**Poids et apport calorique:** nous remarquons l’absence d’effet significatif du jeûne du mois de Ramadan sur le poids, avec un faible taux d’écart diététique comparé à d’autres études [6,12]. Plusieurs études ont noté l’absence d’effet significatif du jeûne sur le poids [14,16]. Certaines études ont retrouvé une réduction significative du poids au cours du jeûne du mois de Ramadan, elle serait secondaire à une baisse importante de l’apport calorique total motivé probablement par le désir du jeûne [20,21]. La préparation au jeûne par des conseils diététiques apportés aux patients qui jeûneent permet d’éviter une prise de poids au cours de ce mois réputé par des repas festifs au Maroc.

**Tension artérielle:** nous avons noté une augmentation significative de la TA diastolique au cours du jeûne. Ce résultat est en désaccord avec les résultats d’autres études qui ont montré l’absence de variation significative de la tension artérielle systolique et diastolique chez des patients hypertendus [20-24], une étude malienne a montré une augmentation significative de la TA au cours du jeûne expliqué par la mauvaise observance du traitement [25]. A noter que dans notre étude seulement 20% des patients sont des hypertendus connus sous traitement.

**Equilibre glycémique:** le risque de déséquilibre glycémique chez le patient diabétique qui jeûnee peut être lié à l’alternance jeûnee diurne et suralimentation festive durant la nuit, ainsi qu’aux modifications du comportement alimentaire et du rythme de vie observées au cours du mois de Ramadan. Dans notre série il n’y avait pas de différence significative entre l’HBA1C à l’inclusion et en post jeûne du mois de Ramadan. Ce qui est en accord avec plusieurs études [8,9,11] rapportant que le jeûnee du Ramadan ne s’est pas accompagné de modifications du taux d’HbA1c, ceci est d’autant plus marqué si les patients sont préparés au jeûne. Toutefois, certaines études [6,8,14,23,26] ont montré une baisse des taux de fructosamine et d’HbA1c après le jeûnee. Une étude a noté une augmentation de l’HBA1C expliquée par la mauvaise observance du traitement [25]. L’analyse en sous-groupe a montré que les facteurs associés au déséquilibre du diabète sont: 1) L’ancienneté du diabète expliqué probablement par les anomalies de la glycorégulation qui sont plus importante 2) L’HBA1C plus élevé à l’inclusion ce qui est en accord avec les résultats de Bouguerra *et al*. [20] ou le jeûne s’est accompagné d’une altération de l’équilibre glycémique chez les patients ayant déjà un mauvais contrôle glycémique avant le jeûne. L´étude de Ouhdouch *et al*. [12] montre au contraire que les patients déséquilibrés ont amélioré leur HBA1C après le jeûne. 3) L’absence d’autosurveillance glycémique au cours du jeûne a influencé négativement l’équilibre glycémique des patients. 4) Le groupe ayant déséquilibré son diabète en post Ramadan a présenté une prise de poids par rapport au groupe sans déséquilibre mais sans que cela soit statistiquement significative. 5) L’âge, le sexe, l’indice de masse corporel n’étaient pas significativement associés au déséquilibre du diabète en post Ramadan.

## Conclusion

Cette étude a permis de montrer qu’en appliquant les recommandations internationales comportant une éducation des patients et un ajustement thérapeutique, tout en tenant compte du degré du risque encouru, le jeûne peut être réalisé avec un risque minime de complications aigues ou de déséquilibre de diabète. Toutefois en pratique clinique courante une consultation au cours du Ramadan (J7) ou à défaut un entretien téléphonique permettrait une correction des erreurs diététiques et un ajustement du traitement en fonction des données de l’autosurveillance si cela est nécessaire.

### Etat des connaissances sur le sujet

- Le jeûne du Ramadan est fortement désiré chez les patients musulmans diabétiques;
- La pratique du jeûne est faite en absence de suivi médicale;
- Le jeûne peut causer des complications aigues du diabète et des complications thromboemboliques.

### Contribution de notre étude à la connaissance

- La classification des patients en niveau de risque permet de mieux expliquer les risques encourus chez les patients;
- L’éducation thérapeutique avant le mois du jeûne et la discussion de la question avant le mois sacré facilite le suivi au cours du jeûne;
- Les ajustements thérapeutiques au début du mois sacré permettent de mieux gérer les complications du jeûne.

### Conflits d’intérêts

Les auteurs ne déclarent aucun conflit d’intérêts.
